# High‐Fat and High‐Sugar Diet Induces Murine Premature Ovarian Failure by Promoting Mitochondrial Oxidative Phosphorylation Response

**DOI:** 10.1002/fsn3.70973

**Published:** 2025-09-18

**Authors:** Kai Li, Yichao Wen, Zeyu Cui, Lele Ling, Chunxia Wang, Bimeng Zhang, Te Liu

**Affiliations:** ^1^ Shanghai Geriatric Institute of Chinese Medicine Shanghai University of Traditional Chinese Medicine Shanghai China; ^2^ Center of Reproductive Medicine Shuguang Hospital Affiliated to Shanghai University of Traditional Chinese Medicine Shanghai China; ^3^ Department of Acupuncture, Shanghai General Hospital Shanghai Jiao Tong University School of Medicine Shanghai China; ^4^ Department of Reproductive Medicine Henan Province Hospital of Traditional Chinese Medicine Henan China

**Keywords:** high‐fat and high‐sugar diet, oxidative phosphorylation signal transduction pathway, premature ovarian failure, single‐cell RNA‐seq

## Abstract

The causes of premature ovarian failure (POF) as a result of high‐fat and high‐sugar (HFHS) diets have not been studied systematically or in depth. In this study, we used single‐cell RNA‐seq (scRNA‐seq) and molecular pathology experimental techniques to systematically analyze the ovarian landscape in HFHS diet‐induced POF in mice. An HFHS diet decreased levels of AMH and E2 and induced a significant amount of follicular atresia in mice, according to the enzyme‐linked immunosorbent assay (ELISA) and pathology results. The scRNA‐seq results also showed that the number of Krt19 + epithelial cell, Csmd1 + cumulus cell, Mgarp + thecal cell, and mt‐CO1 + granulosa cell clusters was much higher in the ovarian tissues of HFHS–POF mice than it was in the control group. According to the KEGG analysis, the differentially transcribed genes in these three cell clusters in different groups were chiefly involved in multiple signal transduction pathways, and their overlapping signaling pathways included oxidative phosphorylation. Immunofluorescence staining and qPCR showed that expression levels of the oxidative phosphorylation signaling pathway were significantly lower in the control group than they were in the thecal cell, epithelial cell, and stromal cell clusters of the HFHS–POF mice. To the best of our knowledge, this study is the first to report the application of scRNA‐seq to analyze the ovarian landscape of HFHS‐fed mice and to verify that the HFHS diet induced POF in mice by activating the oxidative phosphorylation signal transduction pathway in the thecal cell, granulosa cell, and cumulus cell clusters in mouse ovarian tissues.

## Graphical abstract

Our study is the first to report the application of scRNA‐seq to analyze the ovarian landscape of HFHS‐fed mice and to verify that the HFHS diet induced POF in mice by activating the oxidative phosphorylation signal transduction pathway in the thecal cell, granulosa cell, and cumulus cell clusters in mouse ovarian tissues.

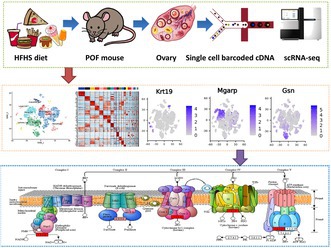

**Abbreviations**
AMH: Anti‐Müllerian hormone
DEGs: Differentially Expressed Genes
ELISA: Enzyme‐linked immunosorbent assay
FSH: Follicle‐stimulating hormone
H&E: Hematoxylin and eosin staining
HDL: High‐density lipoprotein
HFHS: High‐fat and high‐sugar diets
LDL: Low‐density lipoprotein
POF: Premature ovarian failure
scRNA‐seq: Single‐cell RNA‐seq
TC: Total cholesterol
TG: Triglycerides

## 1 Introduction

Amenorrhea, low estrogen levels, high concentrations of gonadotropins, a lack of mature follicles, and infertility are characteristics of premature ovarian failure (POF) in women younger than 40 years old. POF has been tied to metabolic and endocrine disorders and is one of the most common causes of female infertility (Chon et al. 2021; Lin et al. 2020; Rebar 2009). Previous studies have shown that a high‐fat and high‐sugar (HFHS) diet not only affects ovarian function and egg quality (Liu et al. 2005; Liu, Jing, et al. 2021; Liu, Lin, et al. 2021; Rebar 2009; Wang et al. 2014; Wu et al. 2015) but also increases the risk of tumors, obesity, and cardiovascular diseases (Goncalves et al. 2019; Liu, Jing, et al. 2021; Liu, Lin, et al. 2021; Tappy and Le 2010; Wang et al. 2014; Wu et al. 2015; Zmora et al. 2019). Liu and Zhang et al. demonstrated that mice fed on an HFHS diet exhibited significant reproductive dysfunction, including elevated levels of serum testosterone and luteinizing hormone (LH), irregular estrous cycles, and impaired follicular development (Lin et al. 2020; Liu, Jing, et al. 2021; Liu, Lin, et al. 2021). In addition, it was shown that the HFHS diet disrupts amino acid metabolism in the ovaries of mice. In particular, the biosynthesis of ovarian steroid hormones and the metabolism of glucose and lipids were disturbed (Liu et al. 2024). Brookheart et al. used *Drosophila melanogaster* and found that maternal obesity induced by a high‐sucrose diet blocked fertility as a result of disrupted ovarian function. When fed a high‐sucrose diet, female *D. melanogaster* demonstrated a decreased expression of key ovarian mitochondrial regulators and an increased copy number of mitochondrial DNA. These results suggested that the compensatory response to mitochondrial dysfunction was not effective (Brookheart et al. 2017). In addition, we conducted earlier studies and verified that the HFHS diet induced POF, aging, and death of ovarian granulosa cells (OGCs) following deletion of Lin28/YY2 and modification of histone H2A. X site‐specific phosphorylation (Liu, Jing, et al. 2021). Another previous study we conducted also verified that an HFHS diet modified histone H2A. X phosphorylation and activated the DAB2IP/ASK1/p38 signal pathway by blocking the expression of endogenous miR‐146. As a result, OGCs and POF experienced aging (Liu, Lin, et al. 2021). These previous studies, however, have focused only on certain groups of ovarian cells, including OGCs or oocytes. They have not interpreted the effects of the HFHS diet on these cells from the perspective of the ovarian microenvironment.

In this study, we used the single‐cell RNA‐seq (scRNA‐seq) technology to perform an in‐depth analysis of the transcriptomic landscape of multiple cell clusters within the ovarian tissues of an HFHS diet‐induced POF mouse model.

## 2 Materials and Methods

### 2.1 Construction of HFHS–POF Mouse Model

We constructed an HFHS–POF mouse model by referring to the previously published methods (Goncalves et al. 2019; Liu, Jing, et al. 2021; Liu, Lin, et al. 2021). We purchased 10‐week‐old female C57BL/6 mice (*n* = 16) from the Experimental Animal Centre of Shanghai University of Traditional Chinese Medicine (Shanghai, China). Meanwhile, the procedures were followed in accordance with the Helsinki Declaration of 1975, as revised in 2008. We randomly divided the mice into two groups, each with eight mice. We fed mice in the HFHS–POF model group a high‐fat diet (8 g/kg), and they were gavaged with 200 μL of 30% high‐fructose every day for 2 months. We fed the blank control (Ctrl) group a normal diet. The Ethics Committee of the Shanghai Institute of Geriatrics of Traditional Chinese Medicine (SHAGESYDW202309) approved this study. We ensured that the experimental procedures were in compliance with EU Directive 2010/63/EU for Animal Experiments, the National Institutes of Health (NIH) Guide for the Care and Use of Laboratory Animals (NIH Publications No. 8023, revised 1978), and the Laboratory Animal Regulations of the U.K. Animals (Scientific Procedures) Act of 1986, as well as with all associated guidelines.

### 2.2 Lipid Profile

We collected peripheral blood serum from each mouse group. We used chemiluminescence to measure the levels of low‐density lipoprotein (LDL), high‐density lipoprotein (HDL), total cholesterol (TC), and triglycerides (TG), and we followed the manufacturer's instructions.

### 2.3 Enzyme‐Linked Immunosorbent Assay

We collected the peripheral blood from each mouse group, which we then centrifuged for 10 min at 3000 r/min. We collected and analyzed the serum for concentrations of sex hormones (E2, follicle‐stimulating hormone (FSH), and AMH) using an enzyme‐linked immunosorbent assay (ELISA) kit. We prepared and diluted the serum samples using standards, and then added these samples to the ELISA plate. We reacted the samples for 30 min at 37°C and washed the ELISA plate three times. We added an antibody reaction solution and reacted it for another 30 min at 37°C. After we washed the plate three more times, we added chromogenic solutions A and B. Before terminating the reaction, we reacted the resulting solutions for another 30 min at 37°C. We read the OD value within 15 min.

### 2.4 Hematoxylin and Eosin Staining

We used gradient alcohol (75%, 85%, and 95% alcohol and anhydrous alcohol) to dehydrate tissue samples for 4 h. After we immersed the samples in xylene transparent for 1.5 h and paraffin for 4.5 h, we embedded the samples in paraffin blocks. We used xylene to wash the paraffin sections for 10 min and then washed them in an alcohol gradient for 5 min (absolute ethanol, 95%, 85%, and 75%) to complete the deparaffinization. Then, we performed hematoxylin and eosin (H&E) staining, in which the nuclei were stained purplish blue with hematoxylin and the cytoplasm was stained pink with eosin. Next, we mounted the neutral gum and used a microscope to observe the images before collection.

### 2.5 MASSON Staining

We used gradient alcohol (75%, 85%, 95% alcohol, and anhydrous alcohol) to dehydrate the tissue samples for 4 h. We immersed the samples in xylene transparent for 1.5 h and then in paraffin for 4.5 h. We embedded the samples in paraffin blocks. We used a microtome to cut the paraffin tissue wax blocks into 4‐μm‐thick sections. We deparaffinized the samples after we washed them for 10 min in xylene and then washed them for 5 min in each alcohol gradient (absolute ethanol, 95%, 85%, and 75% ethanol). Last, we washed them two more times in a phosphate‐buffered saline (PBS). Subsequently, we subjected the sample to H&E staining using a MASSON kit for 5 min. We washed the sample again, washed with PBS and acidic ethanol differentiation solution for 10 s each. The resulting sample was rewashed with PBS. Subsequently, the sample was reacted with a methylene blue solution for 3 min, washed with PBS, subjected to Ponceau Red staining for 10 min, rewashed with PBS, and subjected to phosphomolybdic acid staining and toluidine blue staining for 5 min each. Next, we washed the sample in 1% glacial acetic acid solution for 1 min, followed by its dehydration and mounting. Last, we used a microscope to observe the acquired images.

### 2.6 Immunofluorescence Staining

We soaked the fresh tissue in 4% paraformaldehyde (Sigma‐Aldrich, St. Louis, MO, USA) at room temperature for 30 min. After we mixed the tissue, we used an ethanol gradient solution to dehydrate the sample and embed it in 4‐μm‐thick paraffin blocks. These sections were deparaffinized by immersing them in xylene. We used an immunohistochemical blocking solution (Beyotime Biotechnology Co. Ltd., Zhejiang, China) to block the resulting tissue sections at 37°C for 30 min. Next, we discarded the blocking solution and used an immunohistochemical washing solution (Beyotime Biotechnology Co. Ltd.) to wash the samples three more times for 5 min at room temperature. We added primary antibodies (Rabbit anti‐MGARP/OSAP Antibody (A09345‐2), Rabbit anti‐Cytokeratin 19/KRT19 Antibody (PB9715), Rabbit anti‐Decorin/DCN Antibody (PB9174), Rabbit anti‐MT‐CO1 Antibody (BA4150), Mouse anti‐COX4I1 Antibody (M05442‐1), Boster Biological Technology Co. Ltd., Wuhan, China) to the sample and incubated the sample for 45 min at 37°C, after which we discarded the antibodies.

We used an immunohistochemical washing solution (Beyotime Biotechnology Co. Ltd.) to wash the resulting sample three times for 5 min at room temperature. Then, we added the following secondary antibodies: DyLight 488 Conjugated AffiniPure Donkey Anti‐mouse IgG (H + L) (BA1145) and DyLight 550 Conjugated AffiniPure Donkey Anti‐Rabbit IgG (H + L) (BA1144) (Boster Biological Technology Co. Ltd., Wuhan, China). We incubated the sample for 45 min at 37°C. After we discarded the antibodies, we washed the resulting sample three more times with an immunohistochemical solution (Beyotime Biotechnology Co. Ltd.) for 5 min at room temperature. Last, to seal the slices, we added an immunofluorescence sealing solution (Sigma‐Aldrich, St. Louis, MO, USA).

### 2.7 scRNA‐Seq (Cell Dissociation, Library Construction, Sequencing, and Data Statistical Analysis)

Experimental personnel performed all scRNA‐seq experiments in the SeekGene Co. Ltd., laboratory. We first washed each group's murine ovary tissues with ice‐cold PBS and minced and enzymatically digested the samples with 25 U/mL collagenase I (Sigma), 25 U/mL DNase I (Worthington, Lakewood, NJ, USA), and 125 U/mL collagenase IV (Sigma) at 37°C, with agitation, for 30 min. After digestion, we used a 40‐μm cell strainer to sieve samples from each group. Then we centrifuged the samples for 5 min at 1500 r/min. We used red blood cell lysis buffer (Solarbio, Beijing, China) to lyse the red blood cells and suspend the precipitated cells. We stained the dissociated single cells using acridine orange/propidium iodide and used a Countstar Fluorescence Cell Analyzer to conduct a viability assessment. We used a MACS dead cell removal kit (Miltenyi Biotec, North Rhine‐Westphalia, Germany) to further enrich the single‐cell suspension (Xu et al. 2022).

We used the SeekOne Digital Droplet Single Cell 3‐library preparation kit (SeekGene) to prepare the scRNA‐seq libraries (Klein et al. 2015; Zheng et al. 2017). We mixed an appropriate number of cells with a reverse transcription reagent and added the sample well to the SeekOne DD Chip S3. Next, we barcoded hydrogel beads and dispensed the partitioning oil into corresponding wells in Chip S3. After we generated an emulsion droplet, we performed reverse transcription for 90 min at 42°C. We inactivated the emulsion for 15 min at 80°C. We used PCR to purify and amplify cDNA from the broken droplet. We then cleaned, fragmented, end‐repaired, A‐tailed, and ligated the amplified cDNA product to a sequencing adaptor. To amplify the DNA representing the 3‐polyA part of the expressing genes, we indexed the PCRs. These products also featured the Cell Barcode and Unique Molecular Index. To clean the indexed sequencing libraries, we used SPRI beads and then used quantitative PCR (KAPA Biosystems KK4824) for quantification and used a DNBSEQ‐T7 platform with the PE100 read length or an Illumina NovaSeq 6000 with a PE150 read length for sequencing. To perform the scRNA‐seq data analysis, SeekGene Co. Ltd. applied the SeekGene Cloud Analysis Platform.

### 2.8 RNA Extraction, Reverse Transcription, and qPCR Detection

We used TRIzol Reagent (Invitrogen) to extract total RNA from each group of cells. To generate cDNA through reverse transcription, we treated total RNA with DNase I (Sigma‐Aldrich) and used the ReverTra Ace‐α First Strand cDNA Synthesis Kit (TOYOBO) for quantification. We used the RealPlex4 RealTime PCR detection system (Eppendorf Co. Ltd., Germany) for qRT‐PCR with SyBR Green RealTime PCR Master MIX (TOYOBO) as a fluorescent dye for nucleic acid amplification. The qPCR amplification cycles followed three steps: (1) denaturation for 15 s at 95°C; (2) annealing for 30 s at 58°C; and (3) extension for 42 s at 72°C. After 40 cycles, we determined the relative expression level of the gene according to the 2‐ΔΔCt calculation method, where ΔCt = Ct_genes—Ct_18sRNA; ΔΔCt = ΔCt_all_groups—ΔCt_blankcontrol_group. For the expression level of 18S rRNA, we corrected the mRNA expression level.

### 2.9 Western Immunoblotting Analysis

Briefly, firstly, each cell of Epithelial Cell, Stromal Cell, and Thecal Cell clusters was isolated and enriched from murine ovaries of Ctrl or HFHS‐POF group by flow cytometry, respectively. The total proteins from each group of cells were electrophoresed using 12% SDS‐PAGE denaturing gel, and then transferred to a PVDF membrane (Millipore). After sealing and washing, the membrane was incubated with primary antibodies (Rabbit anti‐MGARP Antibody (HPA015994, Sigma‐Aldrich), Rabbit anti‐Cytokeratin 19/KRT19 Antibody (PB9715, Boster Biological Technology), Rabbit anti‐Decorin/DCN Antibody (PB9174, Boster Biological Technology), Rabbit anti‐UQCR11 Antibody (MBS839902, MyBioSource), Rabbit anti‐COX4I1 Antibody (VPA00544, Bio‐Rad), Rabbit anti‐Atg5g1 Antibody (PA1‐46178, Thermo Fisher Scientific), Rabbit anti‐*β*‐Actin Antibody (4970, Cell Signaling Technology)) at 37°C and allowed to react for 45 min. After thoroughly washing, the membrane was incubated with second antibodies for a reaction time of 45 min (Goat Anti‐Rabbit IgG H&L (HRP) (ab6721, Abcam)) at 37°C. We washed the membrane with TBST four times at room temperature for 14 min each time. We then used enhanced chemiluminescence (ECL Kit, Pierce Biotechnology) and exposed and developed the film (Sigma‐Aldrich Chemical).

### 2.10 Statistical Analysis

All experiments were completed three or more times. When applicable, the data were given as the mean ± standard error. To evaluate differences, we applied Student's *t*‐test and used a *p* < 0.05 to consider statistical significance.

## 3 Results

### 3.1 HFHS Diet Causes Abnormal Lipid Metabolism, Adversely Affects Sex Hormones, and Induces POF in Mice

**FIGURE 1:**
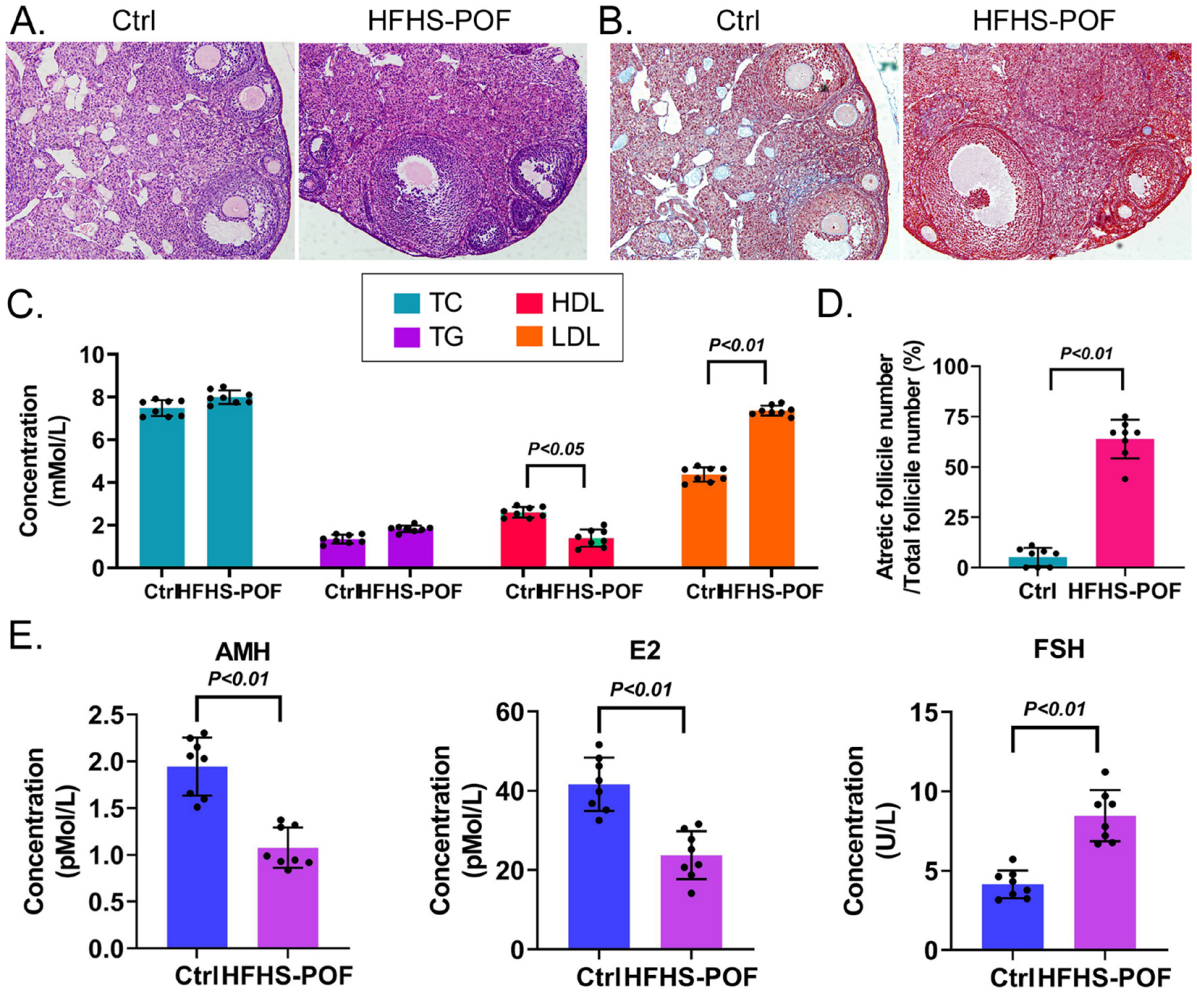
High‐fat, high‐sugar (HFHS) diet‐induced premature ovarian failure (POF) in mice: (A) H&E staining showing the ovarian tissues of the HFHS group mice with the pathological phenomenon POF (200× magnification); (B) Masson staining showing lipid accumulation and atretic follicles in the HFHS group's ovarian tissues (200× magnification); (C) an HFHS diet increased LDL and HDL to abnormal levels in the peripheral blood of the mice model; (D) the atretic follicles the Ctrl group's ovarian tissues were significantly lower than in the HFHS–POF group mice; and (E) ELISA results showing the AMH and E2 levels, which were significantly higher, and the FSH levels, which were significantly lower, in the peripheral blood of the Ctrl group than in the HFHS–POF group.

Dark red staining indicated the ovarian tissues of the HFHS–POF group. Extensive lipid deposition in these tissues was revealed by the pathological diagnostic results of H&E staining, which showed that the Ctrl mice group mice had a loose ovarian tissue structure. Although the granulosa cell morphology was normal, it was more common to find normal follicles. In the HFHS–POF group, however, the granulosa cell staining was darker, which showed that the ovary was atrophied. In this group, the interstitial void was smaller, and atretic follicles were more common (Figure 1A). According to MASSON staining, blue collagen covered the ovarian tissue space in the Ctrl group. The overall staining of the ovary was light red. Follicles could be seen at all levels. The ovarian tissue of the HFHS–POF group was stained dark red because of a large amount of lipid deposition. We did not observe any blue staining in the interstitial part of the ovary of the HFHS–POF group, which suggested a severe loss of collagen (Figure 1B). According to the blood lipid analysis, the peripheral blood of the HFHS–POF mice showed significantly lower HDL and significantly higher LDL than the Ctrl group mice (Figure 1C). The proportion of atretic follicles in the Ctrl group was significantly lower than in the HFHS–POF group (Figure 1D). According to the ELISA results, the AMH and E2 levels in the peripheral blood of the HFHS–POF group were significantly lower, and the levels of FSH were significantly higher than in the Ctrl group (Figure 1E). These findings suggested that an HFHS diet not only led to abnormalities in lipid metabolism and sex hormones but also induced ovarian tissue damage and POF in mice.

### 3.2 Overview of Multiple Cell Clusters in the Ovarian Tissue of Normal and HFHS–POF Mice

**FIGURE 2:**
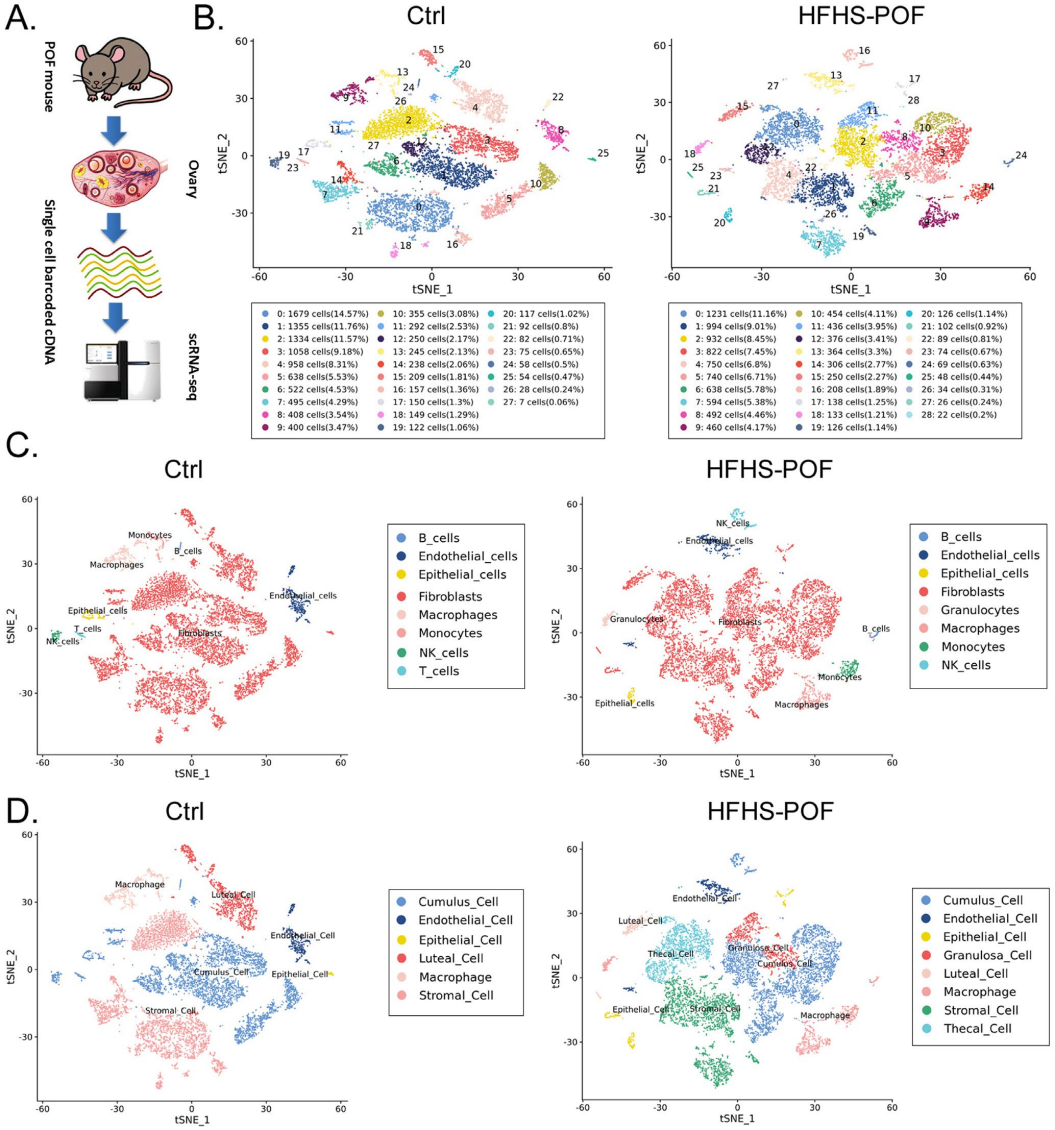
Overview of multiple cell clusters in the ovarian tissues of normal and high‐fat, high‐sugar diet‐induced premature ovarian failure (HFHS–POF) mice: (A) Experimental route of single‐cell RNA‐seq (scRNA‐seq); (B) clustering and visualization analysis of scRNA‐seq data; (C) clustered annotation analysis of scRNA‐seq raw reference dataset using the SingleR tool for cell‐type identification based on reference datasets; and (D) calibration of the scRNA‐seq raw reference dataset with classical ovarian cell cluster biomarkers.

To investigate the effects of the HFHS diet on the clusters of each cell in mouse ovarian tissues, we collected ovarian tissues from both the Ctrl and HFHS–POF groups for scRNA‐seq assay (Figure 2A). First, we clustered and visualized the scRNA‐seq data using Seurat software (Butler et al. 2018). We conducted several analyses, including *t*‐distributed stochastic neighbor editing (*t*‐SNE) dimensionality reduction, clustering, and biomarker gene recognition. The results showed that the ovary of the Ctrl group mice had 28 cell clusters and the ovary of the HFHS–POF group mice had 29 cell clusters (Figure 2B). Further, we analyzed this raw reference dataset for cluster annotation using the SingleR tool to identify cell types based on reference datasets (Aran et al. 2019). We annotated these cell clusters as the cell type with the highest correlation to the reference dataset by identifying the relationship between the cell expression profile to be identified and the single‐cell reference expression profile dataset. The analysis results showed that a total of 11,527 cells (100%) were obtained from 28 cell clusters in the ovaries of the Ctrl group. These cell clusters can be classified as follows: B cells (58 cells, 0.5%), Endothelial cells (490 cells, 4.25%), Epithelial cells (157 cells, 1.36%), Fibroblasts (9952 cells, 86.34%), Macrophages (428 cells, 3.71%), Monocytes (245 cells, 2.13%), NK cells (122 cells, 1.06%), and T cells (75 cells, 0.65%). A total of 11,034 cells (100%) were obtained from 29 cell clusters in the ovarian tissues of HFHS–POF mice, which can be classified as follows: B cells (69 cells, 0.63%), Endothelial cells (486 cells, 4.4%), Epithelial cells (126 cells, 1.14%), Fibroblasts (9246 cells, 83.8%), Granulocytes (133 cells, 1.21%), Macrophages (460 cells, 4.17%), Monocytes (306 cells, 2.77%), and NK cells (208 cells, 1.89%) (Figure 2C). Finally, we calibrated these cell clustering results by combining the classic ovarian cell cluster biomarkers with multiple current research reports. The cluster analysis results showed that the ovaries of the Ctrl group contained Cumulus Cell (4882 cells, 42.35%), Endothelial Cell (490 cells, 4.25%), Epithelial Cell (54 cells, 0.47%), Luteal Cell (1312 cells, 11.38%), Macrophages (645 cells, 5.6%), and Stromal Cell (4144 cells, 35.95%). The ovaries of the HFHS–POF group contained Cumulus Cell (3816 cells, 34.58%), Endothelial Cell (438 cells, 3.97%), Epithelial Cell (366 cells, 3.32%), Granulosa Cell (928 cells, 8.41%), Luteal Cell (298 cells, 2.7%), Macrophages (968 cells, 8.77%), Stromal Cell (2613 cells, 23.68%), and Thecal Cell (1607 cells, 14.56%) (Figure 2D). In addition to these major subpopulations, a small number of cell clusters were also present that have not yet been annotated. Significant differences in the number and distribution of cell clusters in the ovarian tissues of both HFHS–POF and normal mice were revealed by the cluster analysis.

### 3.3 Overview of the Expression of Biomarker Genes in the Ovarian Tissues of Normal and HFHS–POF Mice in Each Cluster

**FIGURE 3:**
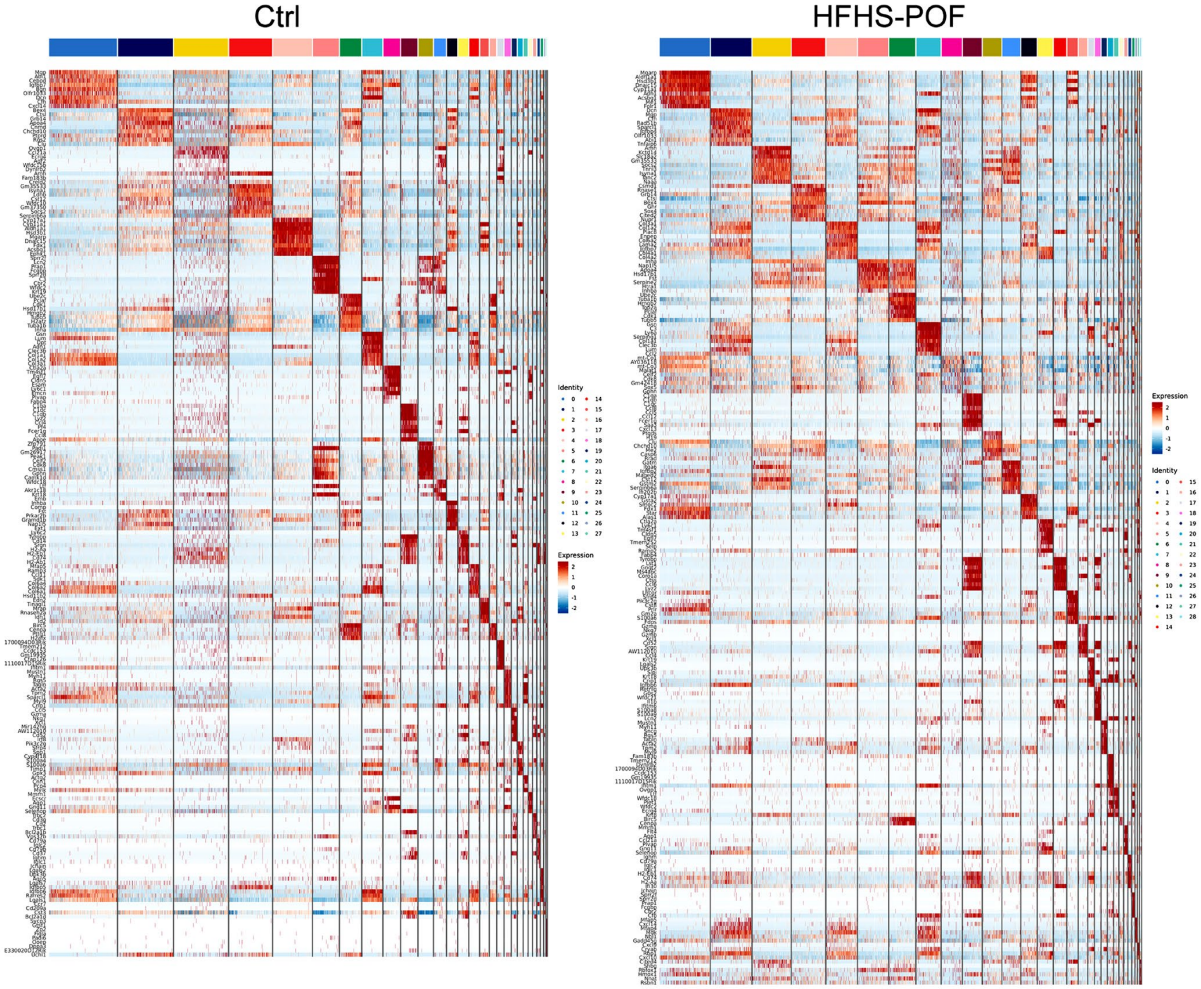
Biomarker gene expression in mouse ovarian tissue clusters.

All differentially expressed genes (DEGs) with a fold change of Log2FC ≥ 0.25 of each cluster were identified based on Avg_log2FC. First, the values of Log10 (UMI + 1) were normalized and clustered. The cluster analysis results showed that the ovarian tissues of the Ctrl group corresponded to 3# (Cumulus Cell), 8# (Endothelial Cell), 25# (Epithelial Cell), 15# (Luteal Cell), 9# (Macrophage), and 7# (Stromal Cell) cells in the ovarian tissues of the HFHS–POF group. Key biomarker genes, such as Amh, Ctla2a, Lgals2, Edn2, C1QA, GSN, and DCN, were significantly expressed in both the Ctrl and HFHS–POF mice (Figure 3). In the ovarian tissues of the HFHS–POF mice, they corresponded to 3# (Cumulus Cell), 13# (Endothelial Cell), 17# (Epithelial Cell), 15# (Luteal Cell), 9# (Macrophage), 7# (Stromal Cell), 0# (Thecal Cell), and 8# (Granulosa Cell). Key biomarker genes, such as Csmd1, Ctla2a, Krt19, Lhcgr, C1qa, Gsn, Dcn, Mgarp, and mt‐CO1, were significantly expressed in both the Ctrl group and HFHS–POF group mice (Figure S3).

**FIGURE 4:**
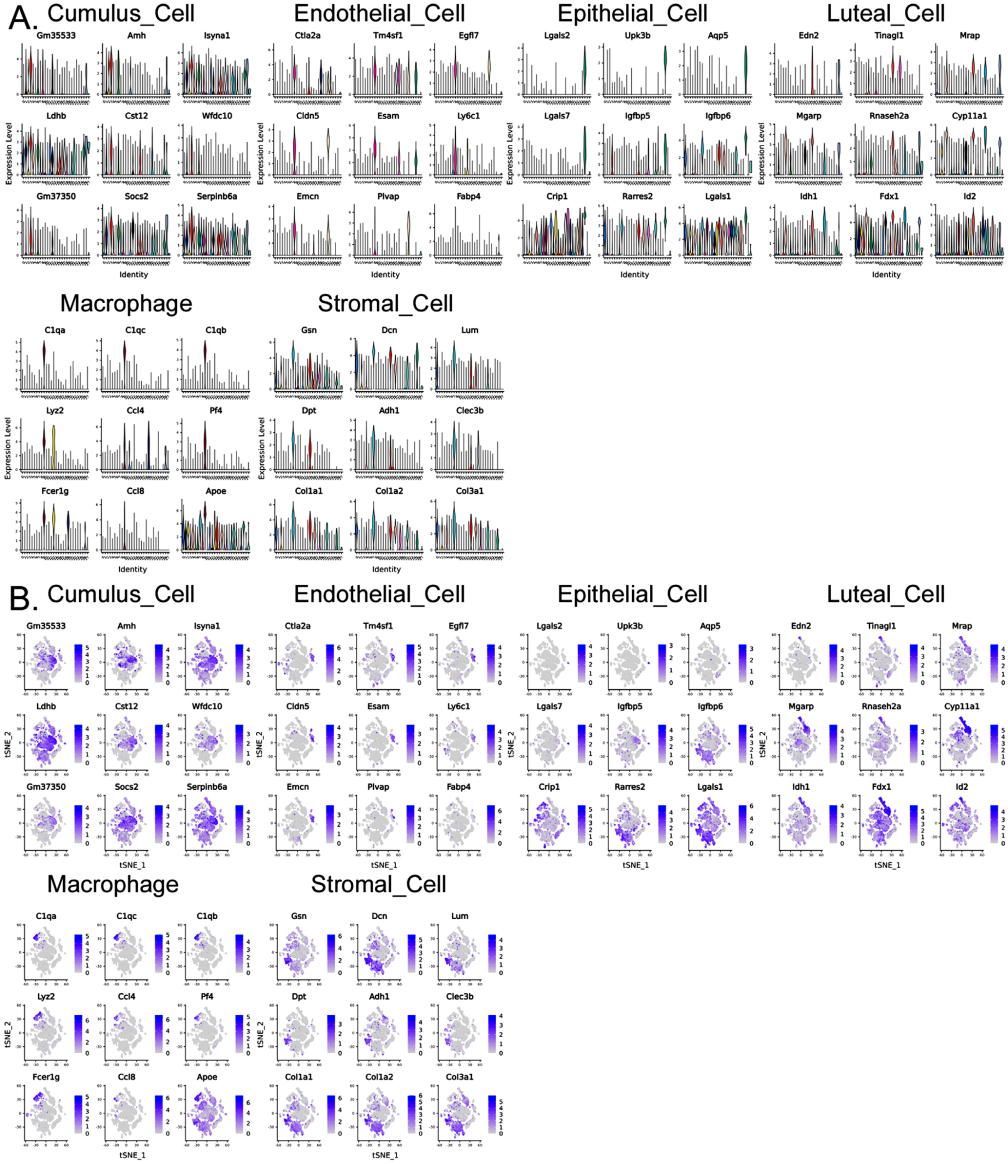
Control (Ctrl) biomarker gene results of a cellular cluster in the ovarian tissue of mice: (A) Expression of the top nine genes in a violin diagram, and (B) expression of *t*‐SNE dimensionality of the top nine genes in different cell clusters.

**FIGURE 5:**
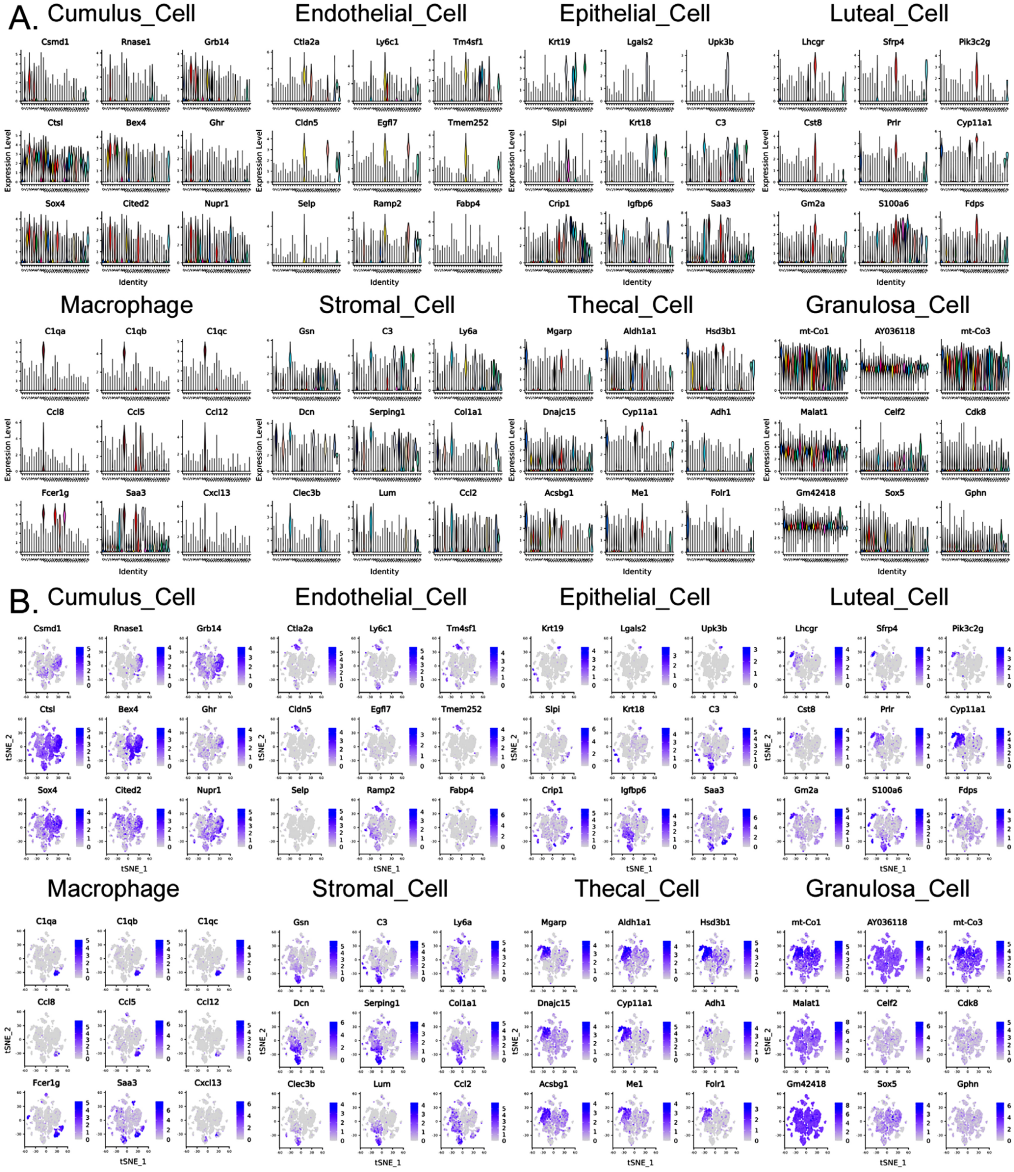
Premature ovarian failure (HFHS–POF) biomarker gene results of a cellular cluster in the ovarian tissue of mice: (A) Expression of the top nine genes in a violin diagram, and (B) expression of *t*‐SNE dimensionality reduction of the top nine genes in different cell clusters.

The *t*‐distribution neighborhood embedding algorithm, also known as *t*‐SNE, is a dimensionality reduction and subtraction algorithm. Typically, this algorithm is employed to project the sequencing dataset into multiple dimensions and clusters. Using *t*‐SNE, we placed similar cell clusters together to specifically label the expression of a single key biomarker gene in each cell cluster. The study results showed that the *t*‐SNE typing results were consistent with those of the previous analysis (Figures 4 and 5, Table S1). The *t*‐SNE typing results also suggested that even in the same cell cluster type, the expression of key biomarker genes was significantly different for different groups of mouse ovarian samples obtained from different sources.

### 3.4 Significant Differences in the Number of Clusters and Gene Transcription Landscape of Epithelial Cell, Stromal Cell, and Thecal Cell Ovary Tissues in Mouse Ovaries

**FIGURE 6:**
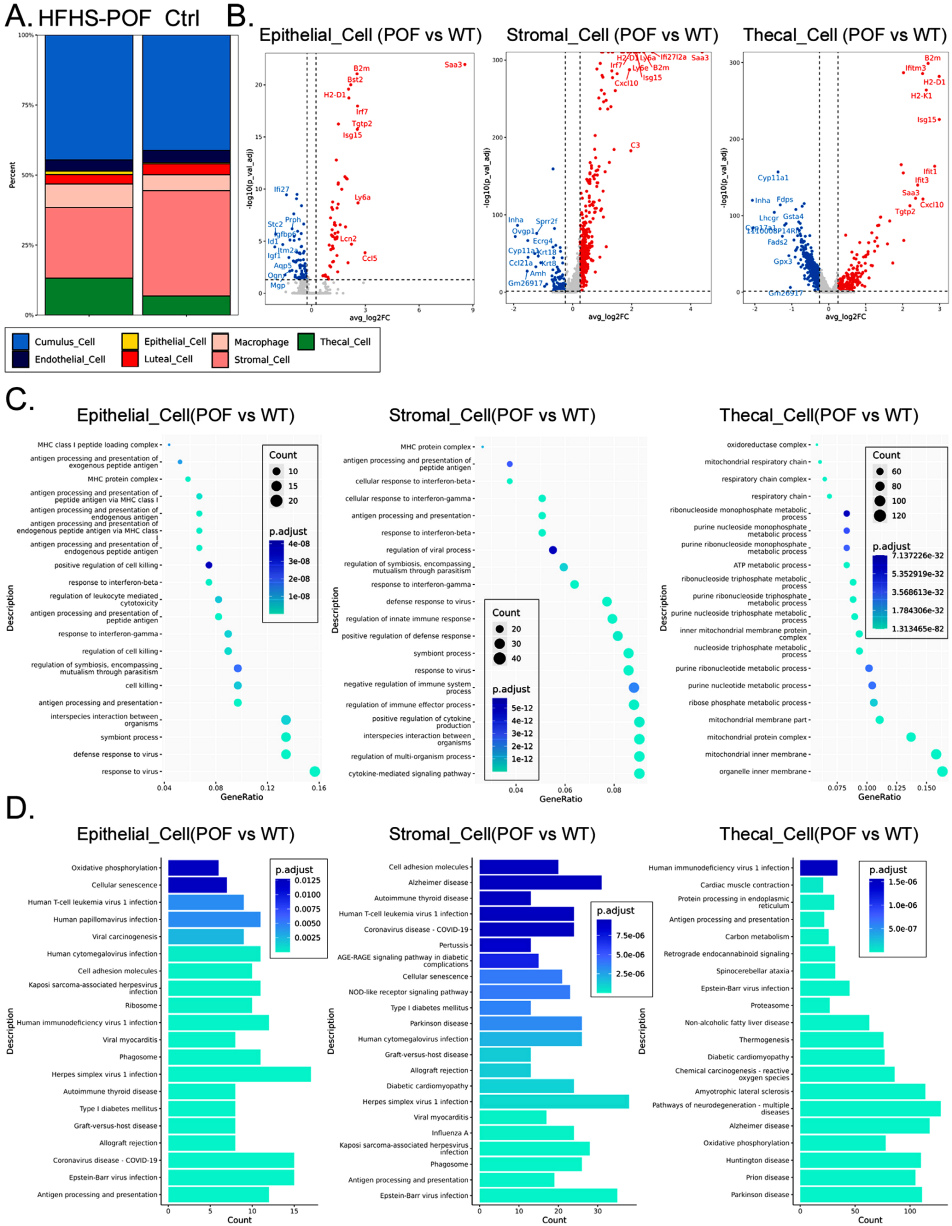
Analysis of the number of Epithelial Cell, Stromal Cell, and Thecal Cell clusters and their gene transcription landscape: (A) Number of Epithelial Cell and Thecal Cell clusters in the ovarian tissues of high‐fat, high‐sugar diet‐induced premature ovarian failure (HFHS–POF) mice was significantly lower in the HFHS–POF group than in the Ctrl group, whereas the number of Stromal Cell clusters was significantly higher in the Ctrl group than in the HFHS–POF group; (B) differentially transcribed genes in three cell clusters in a volcano map; and (C) GO prediction and (D) KEGG prediction of differentially transcribed genes in three cell clusters.

To determine the number of cells in the Epithelial Cell and Thecal Cell clusters in the ovarian tissues of the HFHS–POF mice, we analyzed the inherent cell clusters of the ovarian tissue. We found significantly higher numbers in the Epithelial Cell and Thecal Cell clusters than in the Ctrl group. In the HFHS–POF mice, however, the number of cells in the Stromal Cell cluster was significantly lower (Figure 6A), and the number of cells in the Cumulus Cell was slightly higher than in the Ctrl group. The expression levels of the Bst2, B2m, Cxcl10, H2‐D1, Irf7, Isg15, Ly6a, and Saa3 genes were significantly lower in the HFHS–POF mice than in in the Ctrl group. Conversely, the expression levels of the Cyp11a1, Inha, and Gm26917 genes in these three cell clusters were significantly higher in the Ctrl group than in the HFHS–POF mice (Figure 6B).

According to the gene ontology (GO) analysis, results showed that the differentially transcribed genes in the three cell clusters Epithelial Cell, Stromal Cell, and Thecal Cell were involved in multiple biological processes (Figure 6C). The Epithelial Cell cluster was involved in interspecies interaction between organisms (BP), amide binding (MF), and cytosolic part (CC) as well as in various other processes. The Stromal Cell cluster was involved in such processes as cytokine‐mediated signaling pathway (BP), collagen‐containing extracellular matrix (CC), and cytokine receptor binding (MF). The Thecal Cell cluster was involved in ribose phosphate metabolic process (BP), organelle inner membrane (CC), and peptide binding (MF). We did not observe any overlapping biological processes among the three cell clusters (Figure 6C). We also performed Kyoto Encyclopedia of Genes and Genomes (KEGG) analysis on the ncRNA‐seq results and filtered out explicitly irrelevant predictions (e.g., virus‐related signaling pathways). The KEGG predictions showed that the differentially transcribed genes in these three cell clusters were involved in multiple signal transduction pathways (Figure 6D). The Epithelial Cell cluster, for example, was involved in cell adhesion molecules, oxidative phosphorylation, and antigen processing and presentation as well as other processes. The Stromal Cell cluster was involved in such processes as pathways of neurodegeneration leading to multiple diseases, oxidative phosphorylation, and chemical carcinogenesis involving reactive oxygen species. The Thecal Cell cluster was also involved in the pathways of neurodegeneration resulting in multiple diseases, amyotrophic lateral sclerosis, and oxidative phosphorylation (Figure 6D).

### 3.5 Significantly Different Expression for the Oxidative Phosphorylation Signal Transduction Pathway on Epithelial Cell, Stromal Cell, and Thecal Cell Clusters in Mouse Ovarian Tissues

**FIGURE 7:**
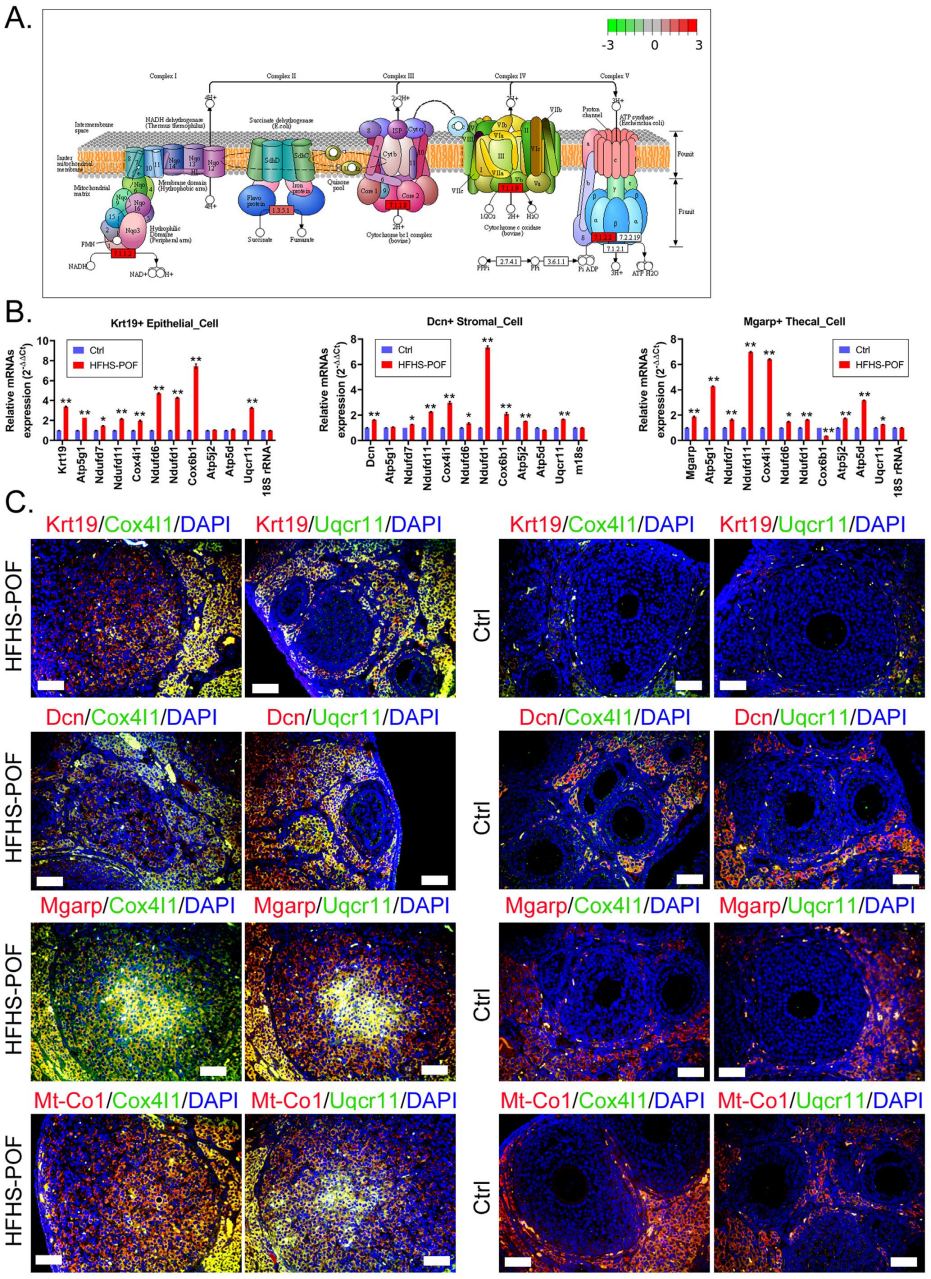
Specific cell clusters in the ovarian tissues of mice in the high‐fat, high‐sugar diet‐induced premature ovarian failure (HFHS–POF) group were highly expressed by factors related to the oxidative phosphorylation signal transduction pathway: (A) Oxidative phosphorylation signaling pathway; (B) the Stromal Cell, Epithelial Cell, and Thecal Cell clusters in the ovarian tissues of HFHS–POF mice were significantly overexpressed the oxidative phosphorylation signal transduction pathway–related genes, as shown by the qPCR results (*p < 0.05 vs. Ctrl group; **p < 0.01 vs. Ctrl group; *t*‐test, *n* = 3); and (C) the Stromal Cell, Epithelial Cell, Thecal Cell, and Granulosa Cell, as well as other cell clusters in the ovarian tissues of HFHS–POF mice, were significantly overexpressed by proteins related to the oxidative phosphorylation signal transduction pathway, as shown by the immunofluorescence assay results.

**FIGURE 8:**
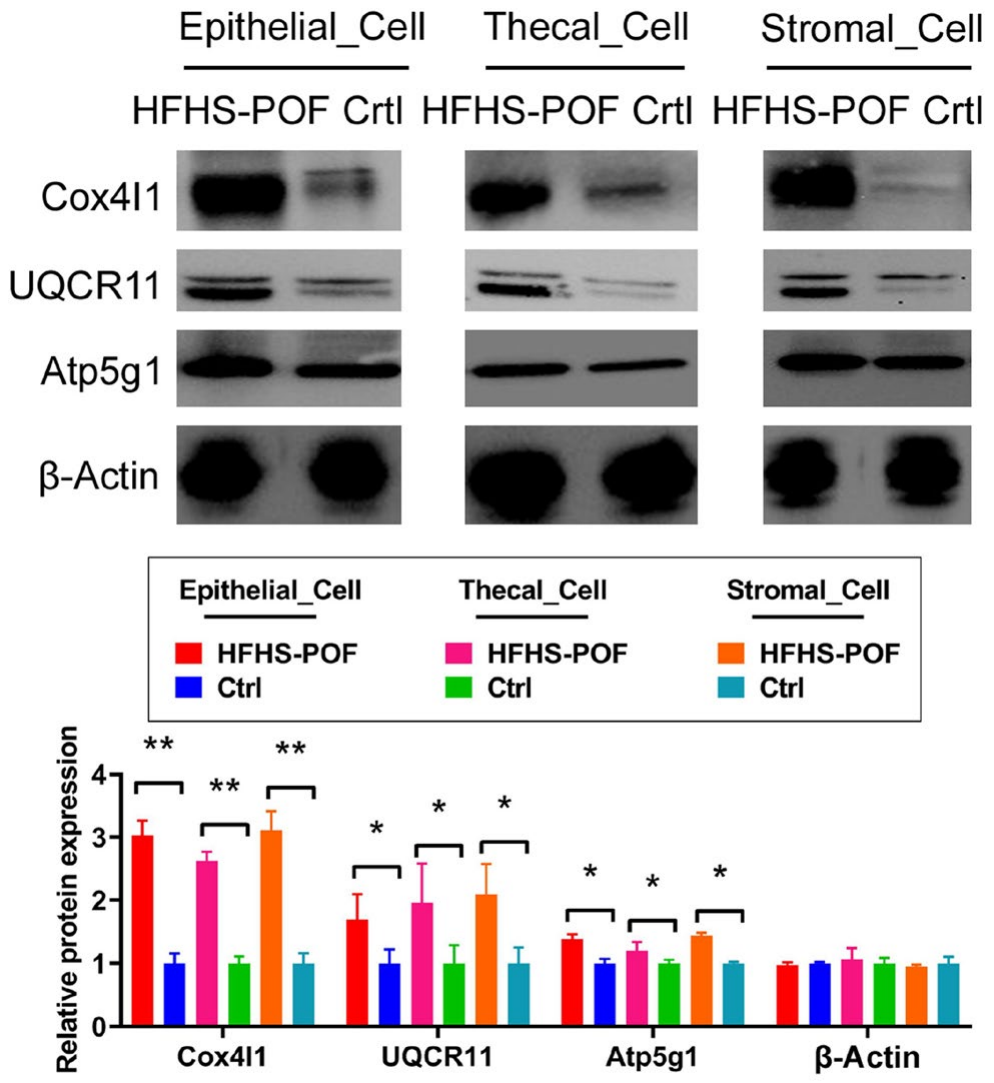
The results of western blot showed that protein expression levels of Cox4I1, Uqcr11, Atp5g1, and Atp5d were significantly lower in the Ctrl group than in the HFHS–POF group. (*p < 0.05 vs. Ctrl group; **p < 0.01 vs. Ctrl group; *t*‐test, *n* = 3).

Intersection analysis of the signaling pathways expressed by Epithelial Cell, Stromal Cell, and Thecal Cell clusters demonstrated that these clusters exhibited differential expression of the oxidative phosphorylation signaling pathway (Figure 7A). All genes on the oxidative phosphorylation signaling pathway were detected through immunofluorescence staining and qPCR analysis of the DEGs in Epithelial Cell, Thecal Cell, and Stromal Cell clusters present in the ovarian tissue of HFHS–POF mice, indicating the presence of the oxidative phosphorylation signal transduction pathway. The mRNA expression levels of Cox4i1, Ndufd1, Ndufd6, Ndufd7, Ndufd11, Uqcr11, and Atp5g1, which are all key genes, were significantly lower in the Ctrl group than in the HFHS–POF group (Figure 7B). The immunofluorescence staining results demonstrated that the Cox4i1 and Uqcr11 protein expression levels in the Epithelial Cell, Stromal Cell, and Thecal Cell clusters of the ovarian tissue of the Ctrl group were significantly lower than those of the HFHS–POF mice (Figure 7C). Meanwhile, the results of western blot showed that protein expression levels of Cox4I1, Uqcr11, and Atp5g1 were significantly lower in the Ctrl group than in the HFHS–POF group (Figure 8). These results indicated that the oxidative phosphorylation signaling pathway was expressed in multiple cell clusters in the ovarian tissue as a result of an HFHS diet.

## 4 Discussion

It has been reported that an HFHS diet not only increases the risk of obesity, cancer, and cardiovascular diseases (Goncalves et al. 2019; Liu, Jing, et al. 2021; Liu, Lin, et al. 2021; Tappy and Le 2010; Wang et al. 2014; Wu et al. 2015; Zmora et al. 2019) but also severely affects ovarian function and oocyte quality (Liu et al. 2005; Liu, Jing, et al. 2021; Liu, Lin, et al. 2021; Rebar 2009; Wang et al. 2014; Wu et al. 2015). Although some in‐depth studies on the molecular biological mechanism of HFHS diet‐induced ovarian insufficiency are available, comprehensive data on a range of factors is still lacking, including the ovarian microenvironment and gene transcription in cellular subpopulations. In this study, we revealed the effects of HFHS diets on the transcriptomic landscape of multiple cell subsets in the mouse ovarian microenvironment using scRNA‐seq. According to the results of the scRNA‐seq analysis, the number of Csmd1 + Cumulus Cell, Krt19 + Epithelial Cell, Mgarp + Thecal Cell, and mt‐Co1 + Granulosa Cell clusters in the ovarian tissues of the Ctrl group was significantly lower than that of the HFHS group mice. The number of Gsn+/Dcn + Stromal Cell clusters, however, was significantly higher than that of the HFHS group mice. These results, combined with the pathological analysis, indicated that the interstitial parts of the ovarian tissues were closely arranged in the HFHS group mice. The mesenchymal cells and the epithelioid cells shared the same phenotype. These pathological test results aligned with the scRNA‐seq results—that is, the number of Epithelial Cell clusters increased and the number of Stromal Cell clusters decreased. Hence, we hypothesized that the HFHS diet induced the transformation of mouse mesenchymal cells into epithelial cells (i.e., MET). Alteration of epithelioid cells in the loose connective tissue of the ovarian medullary region caused the entire ovarian tissue to stiffen and constrict, which was not conducive to the efflux of mature oocytes.

Next, we focused on the differential changes in three other cell clusters in the microenvironment of ovarian tissues, namely Thecal Cell, Granulosa Cell, and Cumulus Cell. The scRNA‐seq analysis showed that the number of these three cell clusters tended to increase. These results should be explored further. Research results indicated that the number of granulosa cells, thecal cells, and cumulus cells decreased significantly in the ovaries of patients or POF animal models as a result of chemotherapy drugs, infection, and chronic inflammation. These previous studies found that these issues resulted in a persistent decrease in atretic follicle accumulation as well as the number of normal mature follicles (Chon et al. 2021; Liu et al. 2005; Rebar 2009; Wang et al. 2014). However, contrary to the current report, the number of these three cell clusters in the ovaries of HFHS diet‐induced POF mice significantly increased. We believe that our scRNA‐seq results reflect the distinct properties of HFHS–POF. The metabolic network of steroid hormones requires steroids as raw materials. The intake of an HFHS diet dysregulates the steroid hormone metabolism, which affects the number and activity of cell subsets closely related to the production of sex hormones. The development of follicles in mammalian ovaries depends on the regulation of related hormones. For example, pituitary gonadotropins (e.g., FSH and LH) stimulate the development of primitive follicles, which produce large amounts of estrogen. Estrogen is synthesized synergistically by the granulosa and thecal cells of the ovary under the action of pituitary gonadotropin. The androgen synthesized by thecal cells then enters granulosa cells through the basal membrane and is converted into estrogen. Thecal cells form the outer cells of the follicle and maintain the follicle structure integrity, develop OGCs and oocytes in the microenvironment, and provide substrates to produce steroid hormones. The important role played by thecal cells thus regulates the reproductive endocrine system. However, several studies have shown that an abnormal increase in the number or activity of thecal cells is important for investigating the induction of hormone‐related gynecological diseases (e.g., polycystic ovary syndrome, ovarian cancer, and follicular membrane cell tumors). As in the pathological manifestations of polycystic ovary syndrome, patients will develop high androgen (persistently high free testosterone) levels and ovarian insufficiency. Therefore, an increase in the levels of thecal cells in the ovarian tissues of HFHS–POF mice does not provide an equal amount of estrogen for follicle maturation. On the contrary, such an increase in their number will further increase the level of androgens, leading to hormonal endocrine disorders and impairing the normal development of follicles.

Interestingly, HFHS diets significantly increased the expression of the oxidative phosphorylation signaling pathway in all three cell clusters: Thecal Cell, Granulosa Cell, and Cumulus Cell. Oxidative phosphorylation is a crucial biochemical process in the cell and the ultimate metabolic pathway for cellular respiration (Boneh 2006; Huttemann et al. 2007; Nolfi‐Donegan et al. 2020; Vander Heiden et al. 2009). After glycolysis and the tricarboxylic acid cycle, this process causes “energy currency” ATP to be produced (Boneh 2006; Huttemann et al. 2007; Nolfi‐Donegan et al. 2020; Vander Heiden et al. 2009). Thus, oxidative phosphorylation is needed to maintain normal physiological cell function (Boneh 2006; Huttemann et al. 2007; Nolfi‐Donegan et al. 2020; Vander Heiden et al. 2009). We hypothesized that excessive glycolipid intake through the HFHS diet provides ample raw materials for oxidative phosphorylation and catalyzes the overactivation of its reactions. Oxidative phosphorylation is one of the main sources of intracellular ROS (Boneh 2006; Huttemann et al. 2007; Nolfi‐Donegan et al. 2020; Vander Heiden et al. 2009). The inner mitochondrial membrane includes ROS at the substrate end of the respiratory chain. As the mitochondria's electron‐transport chain transfers electrons to O_2_, some of that O_2_ forms O_2_^−^ or H_2_O_2_ (Boneh 2006; Huttemann et al. 2007; Nolfi‐Donegan et al. 2020; Vander Heiden et al. 2009). Because it is the precursor for most ROS, O_2_^−·^ is the most important electron. Protease complexes I and III produce this electron in the respiratory chain of the inner mitochondrial membrane. This part of ROS, which is a metabolic by‐product, has long been regarded as a toxic molecule that damages biological macromolecules. Therefore, the HFHS diet not only activates oxidative phosphorylation to produce more ATP for ovarian cells but also produces more toxic ROS, causing cell damage. This may be one of the potential mechanisms through which HFHS diets induce damage in ovarian tissue.

## 5 Conclusions

**FIGURE 9:**
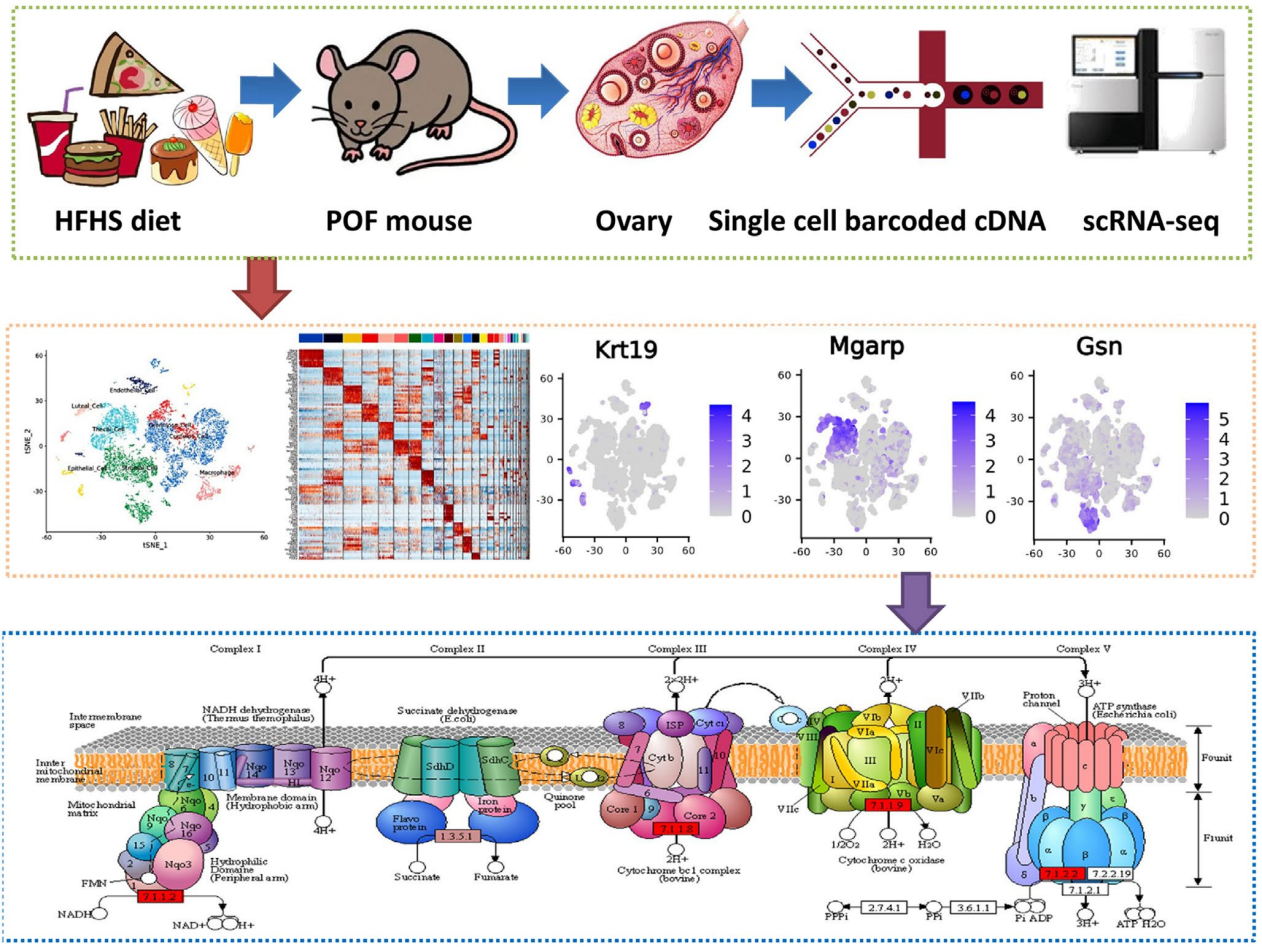
Single‐cell RNA‐Seq (scRNA‐seq) investigation of the ovarian landscape of HFHS‐fed mice.

In this study, we used scRNA‐seq to investigate the ovarian landscape of HFHS diet‐fed mice. The results confirmed that an HFHS diet activated the oxidative phosphorylation of three cell clusters in mouse ovarian tissues: Thecal Cell, Granulosa Cell, and Cumulus Cell. These results also demonstrated that the phosphorylation signaling pathway induced POF in mice (Figure 9).

## Author Contributions

Kai Li, Yichao Wen, Zeyu Cui, Lele Ling, and Te Liu performed the majority of the experiments in the study. Te Liu, Chunxia Wang, and Bimeng Zhang contributed to the analysis of experimental data. Te Liu, Chunxia Wang, and Bimeng Zhang contributed to the study design, manuscript writing, and provided experimental funding support. All authors commented on previous versions of the manuscript. All authors read and approved the final manuscript.

## Conflicts of Interest

The authors declare no conflicts of interest.

## Supporting information


**Figure S3.** fsn370973‐sup‐0001‐Figure3S.jpg.

**Table S1.** fsn370973‐sup‐0002‐Table1S.xlsx.

## Data Availability

The data that support the findings of this study are available from the corresponding author upon reasonable request.
