# Supplementary figures and images for: High‐Fat and High‐Sugar Diet Induces Murine Premature Ovarian Failure by Promoting Mitochondrial Oxidative Phosphorylation Response
Source: Food Sci Nutr. 2025 Sep 18;13(9):e70973. doi: 10.1002/fsn3.70973 (PMC12445113; doi:10.1002/fsn3.70973)

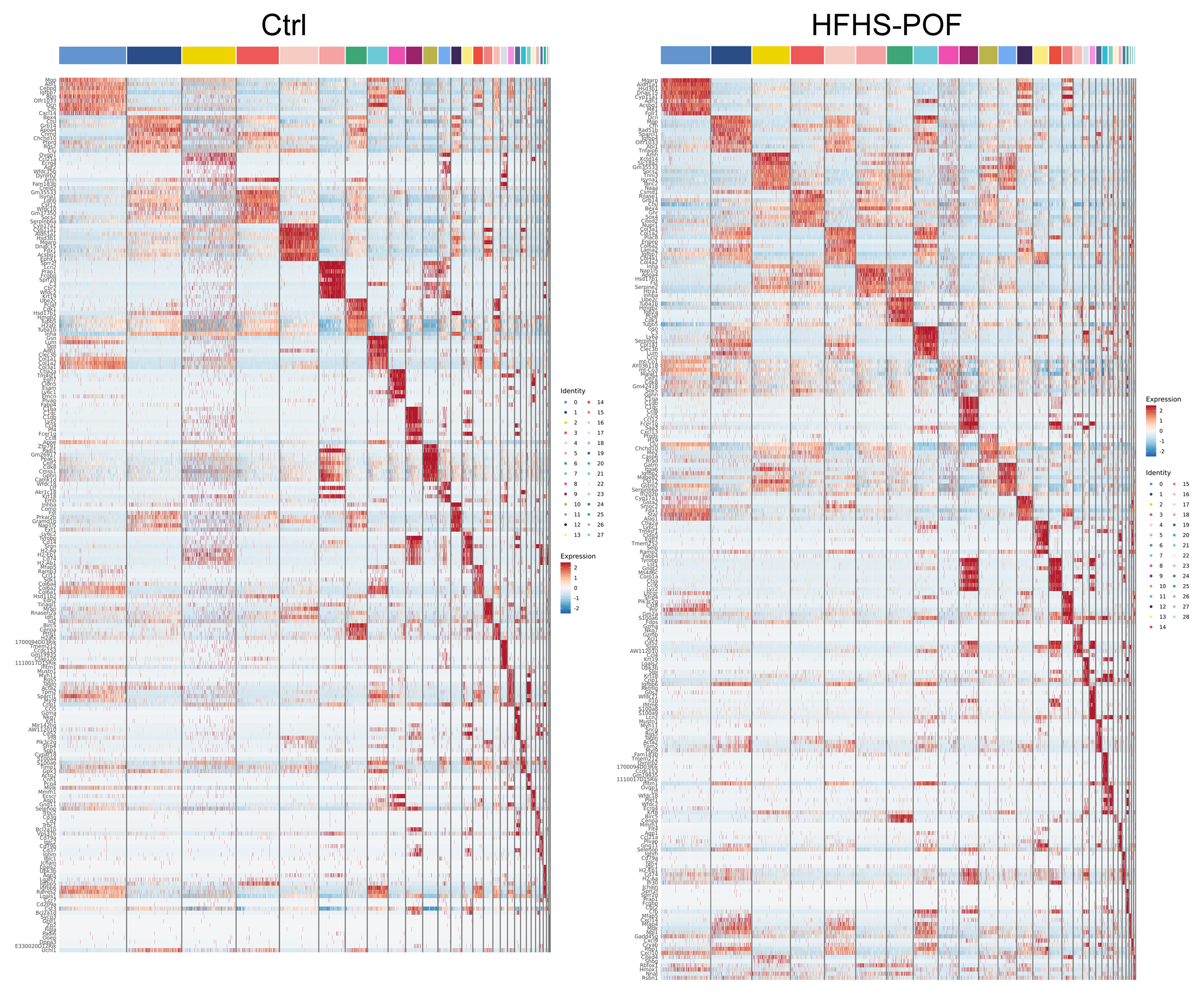

Supplement: Supplementary file 1 — Figure S3. fsn370973‐sup‐0001‐Figure3S.jpg. [file FSN3-13-e70973-s002.jpg]
